# Supplementary material for: The prevalence of symptoms in 24,410 adults infected by the novel coronavirus (SARS-CoV-2; COVID-19): A systematic review and meta-analysis of 148 studies from 9 countries
Source: PLoS One. 2020 Jun 23;15(6):e0234765. doi: 10.1371/journal.pone.0234765 (PMC7310678; doi:10.1371/journal.pone.0234765)
Supplement: S1 Appendix — (DOCX) [file pone.0234765.s002.docx]

**Appendix 1 - Search Strategy**

1. Covid*
2. Coronavirus
3. 2019?nCoV
4. SARS-CoV-2
5. Wuhan pneumonia
6. Symptom*
7. Characteristics
8. Features
9. 1 OR 2 OR 3 OR 4 OR 5
10. 6 OR 7 OR 8
11. 9 AND 10

Limited to 2020
