# Supplementary material for: The prevalence of symptoms in 24,410 adults infected by the novel coronavirus (SARS-CoV-2; COVID-19): A systematic review and meta-analysis of 148 studies from 9 countries
Source: PLoS One. 2020 Jun 23;15(6):e0234765. doi: 10.1371/journal.pone.0234765 (PMC7310678; doi:10.1371/journal.pone.0234765)
Supplement: S9 Fig — (DOCX) [file pone.0234765.s011.docx]

Supplementary Figure 9.
