# Supplementary material for: The prevalence of symptoms in 24,410 adults infected by the novel coronavirus (SARS-CoV-2; COVID-19): A systematic review and meta-analysis of 148 studies from 9 countries
Source: PLoS One. 2020 Jun 23;15(6):e0234765. doi: 10.1371/journal.pone.0234765 (PMC7310678; doi:10.1371/journal.pone.0234765)
Supplement: S1 Table — See the bibliography at the end of the supplementary materials. RT = reverse transcriptase; PCR = polymerase chain reaction. (DOCX) [file pone.0234765.s013.docx]

**Supplementary Table 1.** Study Characteristics. See the bibliography at the end of the supplementary materials. RT = reverse transcriptase; PCR = polymerase chain reaction

| **Study** | **Country** | **N (M:F)** | **Mean age (range)** | **Method of COVID-19 diagnosis** | **Definition of fever** |
| --- | --- | --- | --- | --- | --- |
| WMCHHHPNCI^30^ | China | 2 (1:1) | 38 (unclear) | Real-time RT-PCR | Unclear |
| Chaolin^31^ | China | 3 (unclear) | unclear | Real-time RT-PCR | >37.3^o^C |
| Li^78^ | China | 4 (unclear) | unclear | ≥2 real-time RT-PCR positive tests or a genetic sequence matching 2019-nCoV | >38^o^C |
| Chung^89^ | China | 4 (3:1) | 44 (19-63) | Not described | Not described |
| Chen^100^ | China | 5 (unclear) | unclear | Real-time fluorescent RT-PCR | Not described |
| Wang^111^ | China | 5 (0:5) | 28.8 (25-31) | Real-time RT-PCR | Not described |
| Chang^122^ | China | 13 (10:3) | 34 (34-48) | RT-PCR | Patient reported |
| To^133^ | China | 5 (2:3) | 32.4 (27-41) | Real-time RT-PCR | Not described |
| COVID-19 Australia Team^174^ | Australia | 6 (2:4) | 32 (24-50) | Not described | Not described |
| Yueying^144^ | China | 7 (0:7) | 32.1 (29-34) | Real-time RT-PCR | Fever not reported |
| Li^155^ | China | 9 (5:4) | 36 (15-49) | Real-time RT-PCR | ≥38°C |
| Feng^32^ | China | 9 (0:9) | 29.9 (26-40) | Real-time RT-PCR | ≥37.5°C |
| Liang^43^ | China | 9 (3:6) | 44.2 (22-69) | Not described | Not described |
| Zhang^54^ | China | 10 (6:4) | 53.4 (34-67) | Real-time RT-PCR | Not described |
| Xiabo^65^ | China | 11 (8:3) | 50.36 (unclear) | Not described | Not described |
| Easom^167^ | UK | 11 (10:1) | (26-72) | Real-time RT-PCR | Patient reported |
| Zhao^72^ | China | 11 (4:7) | 44 (18-66) | Real-time RT-PCR | Not described |
| Yu^73^ | China | 11 (3:8) | 53 (32-74) | Real-time RT-PCR | Not described |
| Chan^74^ | China | 12 (7:5) | 62.5 (37-75) | Real-time RT-PCR | Not described |
| Lu^75^ | China | 12 (8:4) | 53 (12 to 68) | Real-time RT-PCR | Not described |
| Zhang^76^ | China | 14 (8:6) | 42.9 (30-72) | Real-time RT-PCR | >37°C |
| Lian^77^ | China | 5805 (unclear) | 47 (unclear) | Real-time RT-PCR | ≥37.3°C |
| Liu^79^ | China | 17 (9:8) | 45 (unclear) | Real-time fluorescent RT-PCR | Fever data not extracted |
| Shi^80^ | China | 18 (9:9) | 47 (31-73) | Not described | Not described |
| Arentz^169^ | USA | 19 (11:8) | 48 (27-56) | PCR | Not described |
| Chen^81^ | China | 20 (10:10) | 37 (unclear) | Real-time RT-PCR | ≥37.5°C |
| Xu^82^ | China | 21 (13:8) | 51 (unclear) | “﻿…positive nucleic acid of the virus and CT scanning of the lung” | ≥37.3°C |
| Yang^83^ | China | 21 (6:15) | 40.9 (unclear) | Real-time RT-PCR | Not described |
| Liu^84^ | China | 21 (11:10) | 70 (43-92) | Real-time RT-PCR | Not described |
| Yang^85^ | China | 21 (17:4) | 56.3 (unclear) | Real-time RT-PCR | Not described |
| Chen^86^ | China | 21 (18:3) | 64 (48-75) | Real-time RT-PCR | Not described |
| Wang^87^ | China | 21 (11:10) | 42 (24-85) | Real-time RT-PCR | Not described |
| Chen^88^ | China | 24 (8:16) | 43 (unclear) | Real-time RT-PCR | Not described |
| Zhang^90^ | China | 25 (11:14) | 51 (25-80) | Real-time RT-PCR | Not described |
| Chu^91^ | China | 28 (15:13) | 42.6 (20-73) | Real-time RT-PCR | Not described |
| Guan^92^ | China | 28 (16:12) | 58 (unclear) | Real-time RT-PCR | ≥37.5°C |
| Deng^93^ | China | 29 (21:8) | 56 (unclear) | Real-time RT-PCR | Not described |
| Wu^94^ | China | 32 (15:17) | unclear | Real-time RT-PCR | Not described |
| Huang^95^ | China | 33 (17:16) | 46 (unclear) | Not described | Not described |
| Kui^96^ | China | 33 (0:33) | 30.5 (24-36) | Real-time fluorescence RT-PCR | Not described |
| Mo^97^ | China | 34 (14:20) | 56.24 (unclear) | “virus nucleic acid detection kit” | Not described |
| Ye^98^ | China | 34 (0:34) | 30 (unclear) | Real-time RT-PCR | Not described |
| Zhou^99^ | China | 35 (0:35) | 61.5 (37-88) | Next-generation sequencing or Real-time RT-PCR | ≥37.3°C |
| Cheng^101^ | China | 36 (25:11) | 69.22 (50-90) | Real-time RT-PCR | Not described |
| Xu^102^ | China | 37 (17:20) | 44.3 (unclear) | RT-PCR | Not described |
| Yu^103^ | China | 40 (15:25) | 48.7 (unclear) | Real-time RT-PCR | Not described |
| Zhang^104^ | China | 41 (30:11) | 49 (unclear) | “Coronavirus antibody” | Not described |
| Wan^105^ | China | 41 (30:11) | unclear | Real-time RT-PCR | ≥37.3°C |
| Wang^106^ | China | 41 (17:24) | 39.1 (unclear) | Real-time RT-PCR | ≥37.3°C |
| Liu^107^ | China | 44 (25:19) | (20-76) | Real-time fluorescence RT-PCR | Not described |
| Huang^108^ | China | 45 (29:16) | 56.7 (unclear) | Real-time RT-PCR and next generation sequencing | ≥37.3°C |
| Xu^109^ | China | 46 (24:22) | unclear | Real-time RT-PCR | ≥37.3°C |
| Chen^110^ | China | 47 (26:21) | 64.9 (unclear) | Real- time RT-PCR | Not described |
| Luo^112^ | China | 47 (28:19) | 62 (unclear) | Real-time RT-PCR | Fever data not extracted |
| Kong^176^ | Korea | 50 (29:21) | unclear | Not defined | Not described |
| Dong^113^ | China | 50 (27:23) | 64 (unclear) | Real-time RT-PCR | Not described |
| Song^114^ | China | 50 (23:27) | 55.2 (22-96) | Real-time RT-PCR | Not described |
| Young^171^ | Singapore | 51 (25:26) | unclear | Real-time RT-PCR | Not described |
| Chen^115^ | China | 51 (25:26) | 49 (16-76) | Real-time RT-PCR | Not described |
| Sun^170^ | Singapore | 51 (32:19) | 45 (16-68) | PCR | Fever data not extracted |
| Jin^116^ | China | 52 (35:17) | 59.7 (unclear) | Real-time RT-PCR | Not described |
| Zhang^117^ | China | 52 (28:24) | 44.5 (unclear) | Real-time RT-PCR | Not described |
| Zhu^118^ | China | 52 (26:26) | 57 (49-69) | “Nucleic acid amplification test” | Not described |
| Zhao^119^ | China | 53 (28:25) | (19-81) | Isolation of SARS-CoV-2 or two positive real-time RT-PCR |  |
| Wu^120^ | China | 54 (36:18) | (26-73) | Real-time RT-PCR | ≥37.3°C |
| Li^121^ | China | 54 (29:25) | unclear | Real-time RT-PCR | Not described |
| Su^123^ | China | 55 (5:50) | (26-58) | Real-time RT PCR | >37.3 |
| Nie^124^ | China | 55 (30:25) | 46.8 (unclear) | Real- time fluorescence RT-PCR | Not described |
| Qi^125^ | China | 56 (12:6) | unclear | Real-time RT-PCR | ≥37.3°C |
| Qi^126^ | China | 57 (31:26) | 46.5 (unclear) | Nucleic acid test positive | ≥38°C |
| Shi^127^ | China | 62 (35:27) | unclear | Real-time RT-PCR | Not described |
| Song^128^ | China | 62 (29:33) | 44.7 (unclear) | Real-time RT-PCR | Not described |
| Sun^129^ | China | 63 (33:30) | (15.2-44.9) | Real-time RT-PCR | Fever data not extraction |
| Tabata^175^ | Japan | 64 (23:41) | 35 (unclear) | Quantitative RT-PCR or nested RT-PCR | Not described |
| Tao^130^ | China | 67 (41:26) | (37-90) | Real-time RT-PCR | Not described |
| Tian^131^ | China | 67 (39:28) | 39.3 (unclear) | Real-time RT-PCR | Not described |
| Wang^132^ | China | 68 (32:36) | 42.5 (0.5-76) | Real-time RT-PCR | Not described |
| Wang^134^ | China | 69 (32:37) | unclear | Real-time RT-PCR | Unclear |
| Xu^135^ | China | 69 (35:34) | unclear | Real-time RT-PCR | Not described |
| Xu^136^ | China | 72 (36:36) | 57.63 (unclear) | Real-time RT-PCR | Not described |
| Xu^137^ | China | 73 (46:27) | unclear | Real-time RT-PCR | Not described |
| Zeng^138^ | China | 75 (42:33) | (16-91) | Information not available | Not described |
| Zhang^139^ | China | 76 (unclear) | unclear | Real-time RT-PCR | Not described |
| Zhang^140^ | China | 78 (39:39) | unclear | Real-time fluorescence RT-PCR | ≥37.3°C |
| Zhang^141^ | China | 80 (39:41) | 46.1 (unclear) | Real-time RT-PCR | Not described |
| Zhang^142^ | China | 80 (34:46) | 53 (26-86) | RT-PCR | Not described |
| Zhang^166^ | UK | 81 (51:30) | 50 (unclear) | Not defined | Not described |
| Zhao^143^ | China | 82 (54:28) | unclear | Real-time RT-PCR | ≥37.3°C |
| Zhou^145^ | China | 83 (44:39) | 45.5 (unclear) | Real-time RT-PCR | Not described |
| Ai^146^ | China | 86 (15:71) | 49 (22-66) | Real-time RT-PCR | Not described |
| Bi^147^ | China | 89 (0:89) | 30.5 (unclear) | PCR | Not described |
| Bian^148^ | China | 89 (30:59) | 33 (unclear) | RT-PCR | ≥37.3°C |
| Cai^149^ | China | 95 (53:42) | (39-58) | Real-time RT-PCR | Not described |
| Cao^150^ | China | 97 (34:63) | (23-82) | Real-time RT-PCR | >37.5°C |
| Chen^151^ | China | 99 (67:32) | 55.5 (21-82) | Not defined | Not described |
| Chen^152^ | China | 101 (56:45) | 44.44 (17-75) | Real-time RT-PCR | Fever data not extracted |
| Chen^153^ | China | 101 (60:41) | (35-100) | Real-time RT-PCR | Not described |
| Chen^154^ | China | 101 (64:37) | 65 (unclear) | Real-time RT-PCR | Not described |
| Chen^156^ | China | 102 (59:43) | 57 (27-85) | RT-PCR | ≥37.3°C |
| Chen^157^ | China | 103 (unclear) | unclear | RT-PCR | ≥37.3°C |
| Chen^158^ | China | 104 (54:50) | (25-93) | Real-time RT-PCR | 37.4-39.1°C |
| Chen^159^ | China | 108 (40:68) | 51 (unclear) | Real-time RT-PCR | 37.4-39.1°C |
| Chen^160^ | China | 109 (59:50) | 55 (unclear) | RT-PCR | ≥37.5° C |
| Chen^161^ | China | 110 (48:62) | unclear | RT-PCR | Not described |
| Chen^162^ | China | 128 (60:68) | (<20 to >65) | RT-PCR | Not defined |
| Cui^163^ | China | 132 (74:58) | 34 (unclear) | Real-time RT-PCR | >37.3 |
| Dong^164^ | China | 135 (72:63) | unclear | RT-dPCR | Fever data not extracted |
| Duan^165^ | China | 136 (66:70) | (25-89) | RT-PCR | Not described |
| Fan^33^ | China | 137 (61:76) | 57 (20-83) | Real-time RT-PCR | Not described |
| Fan^34^ | China | 138 (75:63) | 56 (unclear) | RT-PCR | ≥37.3°C |
| Fei^35^ | China | 140 (71:69) | (25-87) | Not described | Not described |
| Feng^36^ | China | 141 (72:69) | 44 (unclear) | Real-time RT-PCR | ≥37.1°C |
| Feng^37^ | China | 143 (unclear) | unclear | Real-time RT-PCR | Not described |
| Fu^38^ | China | 148 (75:73) | 50 (15-88) | Real-time RT-PCR | Not described |
| Fu^39^ | China | 149 (81:68) | 45.11 (unclear) | Real-time RT-PCR | Not described |
| Fu^40^ | China | 155 (86:69) | unclear | Real-time RT-PCR | ≥37.3°C |
| Gong^41^ | China | 163 (100:63) | 42 (unclear) | Real-time RT-PCR | Not described |
| Gritti^173^ | Italy | 175 (83:92) | 46 (unclear) | Clinical and Radiological assessment | Not described |
| Han^42^ | China | 183 (102:81) | 53.8 (unclear) | Real-time RT-PCR | Not described |
| Huang^44^ | China | 189 (88:101) | 54.25 (33-72) | Real-time RT-PCR | Not described |
| Ji^45^ | China | 191 (119:72) | (18-87) | Nucleic acid test | Not described |
| Kang^46^ | China | 192 (97:95) | (22-87) | Real-time RT-PCR | ≥37.5° C |
| Kluytmans^177^ | Netherlands | 197 (99:98) | 55.94 (18-91) | Real-time RT-PCR | ≥38° C |
| Kujawski^168^ | USA | 200 (99:101) | (<49 to >70) | Real-time RT-PCR | Patient reported or ≥100.4 °F |
| Lei^47^ | China | 201 (128:73) | unclear | Real-time RT-PCR | Not described |
| Lei^48^ | China | 212 (85:127) | 48.5 (17-79) | Real-time RT-PCR | >73.5 |
| Leung^49^ | China | 214 (87:127) | 52.7 (unclear) | Real-time RT-PCR | Not described |
| Li^50^ | China | 221 (108:113) | (20-96) | Real-time RT-PCR | Not described |
| Li^51^ | China | 225 (124:101) | (22-94) | Real-time RT-PCR and next-generation sequencing | ≥37.3°C |
| Li^52^ | China | 236 (110:126) | unclear | Real-time RT-PCR | Not described |
| Li^53^ | China | 238 (138:100) | 55 (unclear) | Real-time RT-PCR | Not described |
| Li^55^ | China | 249 (126:123) | unclear | Real-time RT-PCR | Not described |
| Liang^56^ | China | 265 (unclear) | unclear | Real-time RT-PCR | Not described |
| Liao^57^ | China | 267 (149:118) | unclear | Real-time RT-PCR | Not described |
| Lin^58^ | China | 274 (171:103) | unclear | Real-time RT-PCR | Not described |
| Liu^59^ | China | 291 (145:146) | 46 (unclear) | Real-time RT-PCR | ≥37.3°C |
| Liu^60^ | China | 298 (149:149) | 47 (unclear) | Real-time RT-PCR | Not described |
| Liu^61^ | China | 391 (187:204) | 45 (unclear) | Real-time RT-PCR | Not described |
| Liu^62^ | China | 416 (205:211) | (21-95) |  |  |
| Liu^63^ | China | 425 (240:185) | 56 (unclear) | Real-time RT-PCR | ≥37.5°C |
| Liu^64^ | China | 534 (268:266) | 45 (16-68) | Real-time RT-PCR | Not described |
| Lu^66^ | China | 577 (254:323) | 55 (39-66) | Real-time RT-PCR | Not described |
| Lu^67^ | China | 645 (328:317) | 81.55 (unclear) | Real-time RT-PCR | Not described |
| Luo^68^ | China | 651 (331:320) | 91.23 (unclear) | Real-time RT-PCR | ≥37.5°C |
| Mao^69^ | China | 788 (381:407) | unclear | Real-time RT-PCR | Not described |
| Marchese-Ragona^172^ | Italy | 1099 (640:459) | 59 (35-58) | Not described | Not described |
| Miao^70^ | China | 1590 (911:679) | unclear | Real-time RT-PCR | Not described |
| Nie^71^ | China | 1813 (unclear) | unclear | Real-time RT-PCR | Not described |
