# Supplementary material for: The prevalence of symptoms in 24,410 adults infected by the novel coronavirus (SARS-CoV-2; COVID-19): A systematic review and meta-analysis of 148 studies from 9 countries
Source: PLoS One. 2020 Jun 23;15(6):e0234765. doi: 10.1371/journal.pone.0234765 (PMC7310678; doi:10.1371/journal.pone.0234765)
Supplement: S2 Table — (DOCX) [file pone.0234765.s014.docx]

**Supplementary Table 2.** Meta-analyses of the prevalence of symptoms in adults with laboratory test confirmed COVID-19, subgrouped by country

| **Symptom** | **Country** | **Number of studies** | **Prevalence (95% CI)** | **I^2^** | **Z** | **p-value** |
| --- | --- | --- | --- | --- | --- | --- |
| **Fever** | China | 127 | 80 (77, 83) | 93% | 61 | <0.001 |
|  | UK | 2 | 37 (29, 45) | / | 15 | <0.001 |
|  | Italy | 2 | 78 (59, 93) | / | 10 | <0.001 |
|  | USA | 2 | 55 (37, 72) | / | 8 | <0.001 |
|  | Singapore | 1 | 72 (49, 88) | / | 8 | <0.001 |
|  | Australia | 1 | 49 (47, 51) | / | 100 | <0.001 |
|  | Japan | 1 | 35 (26, 44) | / | 12 | <0.001 |
|  | Netherlands | 1 | 53 (43, 64) | / | 14 | <0.001 |
|  | Korea | 1 | 32 (18, 51) | / | 6 | <0.001 |
|  | **Overall** | **138** | **78 (75, 81)** | **94%** | **59** | **<0.001** |
| **Any cough (dry or productive)** | China | 131 | 58 (54, 61) | 93% | 48 | <0.001 |
|  | UK | 2 | 53 (46, 61) | / | 19 | <0.001 |
|  | Italy | 2 | 52 (32, 72) | / | 7 | <0.001 |
|  | Singapore | 2 | 71 (60, 81) | / | 15 | <0.001 |
|  | USA | 2 | 58 (40, 75) | / | 9 | <0.001 |
|  | Australia | 1 | 71 (69, 72) | / | 130 | <0.001 |
|  | Japan | 1 | 28 (20, 37) | / | 10 | <0.001 |
|  | Netherlands | 1 | 76 (66, 83) | / | 19 | <0.001 |
|  | Korea | 1 | 18 (8, 36) | / | 4 | <0.001 |
|  | **Overall** | **138** | **57 (54, 60)** | **92%** | **49** | **<0.001** |
| **Dry (non-productive) cough** | China | 125 | 57 (54, 61) | 93% | 48 | <0.001 |
|  | UK | 2 | 53 (46, 61) | / | 19 | <0.001 |
|  | Italy | 2 | 52 (32, 72) | / | 7 | <0.001 |
|  | Singapore | 2 | 71 (60, 81) | / | 15 | <0.001 |
|  | USA | 2 | 58 (40, 75) | / | 9 | <0.001 |
|  | Australia | 1 | 73 (48, 89) | / | 7 | <0.001 |
|  | Japan | 1 | 28 (20, 37) | / | 10 | <0.001 |
|  | Netherlands | 1 | 76 (66, 83) | / | 19 | <0.001 |
|  | **Overall** | **136** | **57 (52, 62)** | **93%** | **49** | **<0.001** |
| **Productive cough** | China | 69 | 25 (22, 28) | 99% | 20 | <0.001 |
|  | Singapore | 1 | 24 (15, 37) | / | 7 | <0.001 |
|  | **Overall** | **700** | **25 (22, 28)** | **90%** | **20** | **<0.001** |
| **Dyspnoea** | China | 85 | 23 (18, 28) | 97% | 15 | <0.001 |
|  | UK | 2 | 16 (11, 22) | / | 9 | <0.001 |
|  | USA | 2 | 54 (36, 71) | / | 8 | <0.001 |
|  | Italy | 1 | 71 (50, 86) | / | 8 | <0.001 |
|  | Singapore | 1 | 11 (3, 33) | / | 2 | 0.03 |
|  | Korea | 1 | 4 (1, 18) | / | 1 | 0.16 |
|  | Japan | 1 | 18 (12, 27) | / | 8 | <0.001 |
|  | Netherlands | 1 | 38 (29, 49) | / | 11 | <0.001 |
|  | **Overall** | **94** | **23 (19, 29)** | **97%** | **16** | <0.001 |
| **Wheeze** | China | 15 | 17 (9, 27) | / | 6 | <0.001 |
|  | UK | 1 | 13 (8, 22) | / | 6 | <0.001 |
|  | **Overall** | 16 | 17 (9, 26) | 96% | 6 | **<0.001** |
| **Chest pain** | China | 28 | 6 (3, 10) | 92% | 6 | <0.001 |
|  | Netherlands | 1 | 29 (21, 39) | / | 9 | <0.001 |
|  | UK | 1 | 13 (7, 23) | / | 5 | <0.001 |
|  | **Overall** | **31** | **6, (4, 10)** | **92%** | **6** | **<0.001** |
| **Haemoptysis** | China | 21 | 2 (1, 2) | 69% | 8 | <0.001 |
| **Rhinorrhoea** | China | 30 | 5 (3, 6) | 77% | 10 | <0.001 |
|  | Singapore | 2 | 17 (9, 27) | / | 6 | <0.001 |
|  | Australia | 1 | 28 (26, 29) | / | 71 | <0.001 |
|  | Japan | 1 | 23 (16, 32) | / | 9 | <0.001 |
|  | Korea | 1 | 4 (1, 18) | / | 1 | 0.16 |
|  | Netherlands | 1 | 55 (44, 65) | / | 14 | <0.001 |
|  | UK | 1 | 29 (20, 41) | / | 9 | <0.001 |
|  | **Overall** | **38** | **8 (5, 12)** | **97%** | **10** | **<0.001** |
| **Sore throat** | China | 71 | 10 (9, 12) | 88% | 16 | <0.001 |
|  | Singapore | 2 | 40 (29, 52) | / | 10 | <0.001 |
|  | Japan | 1 | 11 (6, 18) | / | 6 | <0.001 |
|  | Korea | 1 | 32 (18, 51) | / | 6 | <0.001 |
|  | Netherlands | 1 | 40 (30, 50) | / | 12 | <0.001 |
|  | UK | 1 | 57 (46, 68) | / | 13 | <0.001 |
|  | USA | 1 | 8 (1, 35) | / | 1 | 0.15 |
|  | **Overall** | **78** | **12 (10, 14)** | **89%** | **16** | **<0.001** |
| **Myalgia** | China | 67 | 16 (14, 18) | 88% | 23 | <0.001 |
|  | Italy | 2 | 14 (4, 35) | / | 3 | <0.001 |
|  | Korea | 1 | 14 (6, 31) | / | 3 | <0.001 |
|  | Netherlands | 1 | 64 (53, 73) | / | 16 | <0.001 |
|  | UK | 1 | 16 (9, 27) | / | 6 | <0.001 |
|  | **Overall** | **72** | **17 (14, 19)** | **89%** | **22** | **<0.001** |
| **Rigors** | China | 15 | 18 (13, 23) | 90% | 11 | <0.001 |
|  | Korea | 1 | 18 (8, 36) | / | 4 | <0.001 |
|  | USA | 1 | 8 (1, 35) | / | 1 | 0.15 |
|  | **Overall** | **17** | **18 (13, 23)** | **88%** | **12** | **<0.001** |
| **Fatigue** | China | 73 | 31 (27, 35) | 95% | 23 | <0.001 |
|  | Italy | 1 | 19 (8, 40) | / | 3 | <0.001 |
|  | USA | 1 | 42 (19, 68) | / | 4 | <0.001 |
|  | Korea | 1 | 11 (4, 27) | / | 3 | 0.01 |
|  | UK | 1 | 9 (5, 17) | / | 5 | <0.001 |
|  | Netherlands | 1 | 76 (66, 83) | / | 19 | <0.001 |
|  | **Overall** | **78** | **31 (27, 35)** | **96%** | **23** | **<0.001** |
| **Nausea** | China | 26 | 6 (3, 10) | 96% | 5 | <0.001 |
|  | USA | 1 | 8 (1, 35) | / | 1 | 0.15 |
|  | **Overall** | **27** | **6 (3, 10)** | **95%** | **5** | **<0.001** |
| **Vomiting** | China | 25 | 4 (2, 8) | 95% | 5 | <0.001 |
|  | UK | 1 | 3 (1, 10) | / | 2 | 0.03 |
|  | **Overall** | **26** | **4 (2, 8)** | **95%** | **5** | **<0.001** |
| **Diarrhoea** | China | 86 | 10 (8, 12) | 93% | 13 | <0.001 |
|  | Italy | 1 | 24 (11, 45) | / | 4 | <0.001 |
|  | Japan | 1 | 10 (5, 17) | / | 6 | <0.001 |
|  | Netherlands | 1 | 19 (12, 28) | / | 7 | <0.001 |
|  | UK | 2 | 10 (6, 15) | / | 7 | <0.001 |
|  | USA | 1 | 8 (8, 35) | / | 1 | 0.15 |
|  | **Overall** | **93** | **10 (8, 12)** | **93%** | **1** | **<0.001** |
| **Abdominal pain** | China | 17 | 4 (2, 7) | 89 | 6 | <0.001 |
|  | Korea | 1 | 4 (1, 18) | / | 1 | 0.16 |
|  | Netherlands | 1 | 6 (3, 13) | / | 3 | <0.001 |
|  | **Overall** | **19** | **4 (2, 7)** | **88%** | **6** | **<0.001** |
| **Headache** | China | 60 | 10 (9, 12) | 83% | 20 | <0.001 |
|  | Japan | 1 | 17 (11, 26) | / | 8 | <0.001 |
|  | Australia | 1 | 45 (43, 46) | / | 94 | <0.001 |
|  | Korea | 1 | 11 (4, 27) | / | 3 | 0.01 |
|  | Netherlands | 1 | 57 (46, 67) | / | 15 | <0.001 |
|  | UK | 1 | 4 (2, 12) | / | 3 | 0.01 |
|  | USA | 1 | 25 (9, 53) | / | 3 | <0.001 |
|  | **Overall** | **65** | **13 (10, 16)** | **97%** | **12** | **<0.001** |
| **Hypogeusia** | China | 1 | 6 (3, 10) | / | 6 | <0.001 |
|  | Italy | 1 | 83 (44, 97) | / | 4 | <0.001 |
|  | **Overall** | **2** | **4 (1, 8)** | **/** | **4** | **<0.001** |
| **Hyposmia** | China | 2 | 7 (4, 10) | / | 8 | <0.001 |
|  | Italy | 1 | 100 (61, 100) | / | 6 | <0.001 |
|  | **Overall** | **3** | **25 (4, 55)** | **/** | **3** | **<0.001** |
