# Supplementary material for: The prevalence of symptoms in 24,410 adults infected by the novel coronavirus (SARS-CoV-2; COVID-19): A systematic review and meta-analysis of 148 studies from 9 countries
Source: PLoS One. 2020 Jun 23;15(6):e0234765. doi: 10.1371/journal.pone.0234765 (PMC7310678; doi:10.1371/journal.pone.0234765)
Supplement: Attachment — Submitted filename: Response to Reviewers Comments v4 MA MG LG EC LM RW.docx [file pone.0234765.s015.docx]

**Response to Reviewers Comments**

**PONE-D-20-13070**

We thank the reviewers for their compliments and valuable comments, and we are pleased to provide responses below in blue.

**Journal requirements**

Please confirm that you have included all items recommended in the PRISMA checklist including a Supplemental file of the results of the quality assessment for each study assessed and an assessment of publication bias using graphical methods (e.g. Funnel plot) and statistical methods (e.g. Egger’s test) as appropriate.

Thank you – all relevant items, including a PRISMA checklist, are included.

We note that you have indicated that data from this study are available upon request. PLOS only allows data to be available upon request if there are legal or ethical restrictions on sharing data publicly. For information on unacceptable data access restrictions, please see http://journals.plos.org/plosone/s/data-availability#loc-unacceptable-data-access-restrictions.

We are pleased to confirm that the raw extracted data used in this meta-analysis and additional relevant files are freely available online via the Open Science Framework repository (deposited at <https://doi.org/10.17605/OSF.IO/9VX6U>). This information is reflected in the Data Availability section of the methods as below:

“Data Availability

To enable future researchers to expand our work and address its limitations in future iterations, the raw extracted data and additional relevant files are available via an Open Science Framework repository (https://doi.org/10.17605/OSF.IO/9VX6U).”

In your revised cover letter, please address the following prompts:

a) If there are ethical or legal restrictions on sharing a de-identified data set, please explain them in detail (e.g., data contain potentially identifying or sensitive patient information) and who has imposed them (e.g., an ethics committee). Please also provide contact information for a data access committee, ethics committee, or other institutional body to which data requests may be sent.

N/A

b) If there are no restrictions, please upload the minimal anonymized data set necessary to replicate your study findings as either Supporting Information files or to a stable, public repository and provide us with the relevant URLs, DOIs, or accession numbers. Please see http://www.bmj.com/content/340/bmj.c181.long for guidelines on how to de-identify and prepare clinical data for publication.

Please see the above response.

Thank you for stating the following in your Competing Interests section: 'None'. Please complete your Competing Interests on the online submission form to state any Competing Interests. If you have no competing interests, please state "The authors have declared that no competing interests exist.". This information should be included in your cover letter; we will change the online submission form on your behalf.

Thank you – we are pleased to update the title page and covering letter accordingly.

**Reviewer #1**

In this manuscript, Wade and collaborators present the results from a systematic review to identify the prevalence of COVID-19 symptoms worldwide. They pull together 148 studies from 9 countries for a total of 24,410 tested cases and find that fever and persistent cough are the most prevalent symptoms. The study is well conducted, the authors screened several databases including pre-prints services. The statistical analysis is technically sound. However, as the authors correctly acknowledge, the COVID-19 research field is rapidly moving and my main concern is that the search was performed over a month and a half ago and hence may now be outdated. Indeed, very large community surveys (eg https://www.nature.com/articles/s41591-020-0857-9; https://www.nature.com/articles/s41591-020-0916-2 among others) including over 3 mi people of which over 4500 with confirmed infection and over 140,000 with predicted infection have been published since. Given the difference in sample sizes the authors should include this as part of their quantitative analysis or at least mention that other studies have been conducted since April 5th and discuss whether their results/conclusions are still valid or not.

Thank you for highlighting these important publications which we are pleased to include in the discussion.

**Reviewer #2**

I recommend publication of this timely paper.

Thank you

**Editor’s comments**

This is a well conducted piece of work worthy of publication. However, this is a rapidly moving field and the reviewers feel that an update on searches is justified before publication.

We thank the reviewers and Professor Hirst for their kind comments and valuable feedback. COVID-19 research is being published at an unprecedented rate and there are several factors which we feel prevent us from updating the searches, as outlined below:

Systematic reviews typically take years to complete (<http://dx.doi.org/10.1136/bmjopen-2016-012545>) and the median time between searches and publication is 5 months (<https://dx.doi.org/10.1186%2F2046-4053-2-36>). We are proud to have assembled an international team of experts and completed a substantially large and clinically pertinent systematic review within 4 weeks. Between April 5^th^ (when we performed our searches) and May 4^th^ (the date we submitted this work to PLoS) ~1200 additional articles were published/deposited and during the 4 weeks taken for peer review a further ~1800 were published/deposited. We believe that several months would be needed for three independent authors to screen these new ~3000 articles, extract data, resolve disagreements, re-analyse the data and rewrite the manuscript. This demonstrates the scale of the task which we feel is impossible with our current resources and within the specified timeframe. Furthermore, if we were to complete this monumental task, it is probable that thousands of new original articles would be published in this timeframe, which precludes the creation of a perfectly up-to-date systematic review in the midst of the crisis. To mitigate this effect, we have made our original raw data open access for future use. Moreover, we are supporting ongoing work that pools comprehensive regional and national individual patient data from hospitalised COVID-19 patients as well as from population screening and case tracing activities, via the Cochrane Collaboration, Belgian public health institute (Sciensano) and beyond. International networking is being set up with several European countries contributing to this effort. This individual patient data pooling will address the accuracy and predictive value of clinical symptom combinations to predict COVID-19 in the context of the phase of the epidemic.

In short, whilst we fully appreciate the importance of up-to-date searches and would like to be able to undertake this particular revision, we hope that the Editor appreciates the magnitude of such a task and that it is currently out of the realms of possibility.
